# Curcumin Modulates Oxidative Stress, Fibrosis, and Apoptosis in Drug-Resistant Cancer Cell Lines

**DOI:** 10.3390/life12091427

**Published:** 2022-09-13

**Authors:** Sami A. Gabr, Wael M. Elsaed, Mohamed Ahmed Eladl, Mohamed El-Sherbiny, Hasnaa Ali Ebrahim, Saad Mohamed Asseri, Yasir A. M. Eltahir, Nehal Elsherbiny, Mamdouh Eldesoqui

**Affiliations:** 1 Department of Anatomy and Embryology, Faculty of Medicine, Mansoura University, Mansoura 35516, Egypt; 2 Department of Basic Medical Sciences, College of Medicine, University of Sharjah, Sharjah P.O. Box 27272, United Arab Emirates; 3 Department of Basic Medical Sciences, College of Medicine, AlMaarefa University, P.O. Box 71666, Riyadh 11597, Saudi Arabia; 4 Department of Basic Medical Sciences, College of Medicine, Princess Nourah bint Abdulrahman University, P.O. Box 84428, Riyadh 11671, Saudi Arabia; 5 Department of Clinical Medical Sciences, College of Medicine, AlMaarefa University, P.O. Box 71666, Riyadh 11597, Saudi Arabia; 6 Department of Respiratory Care, College of Applied Sciences, AlMaarefa University, Riyadh 11597, Saudi Arabia; 7 Department of Anatomy, Faculty of Medicine, Kordofan University, Elobeid 51111, Sudan; 8 Department of Pharmaceutical Chemistry, Faculty of Pharmacy, University of Tabuk, P.O. Box 741, Tabuk 71491, Saudi Arabia; 9 Department of Biochemistry, Faculty of Pharmacy, Mansoura University, Mansoura 35516, Egypt

**Keywords:** cancer resistance, oxidative stress, apoptosis, curcumin activity, SIRT1 activation

## Abstract

In cancer management, drug resistance remains a challenge that reduces the effectiveness of chemotherapy. Several studies have shown that curcumin resensitizes cancer cells to chemotherapeutic drugs to overcome resistance. In the present study, we investigate the potential therapeutic role of curcumin in regulating the proliferation of drug-resistant cancers. Six drug-sensitive (MCF7, HCT116, and A549) and -resistant (MCF7/TH, HCT116R, and A549/ADR) cancer cell lines were treated with curcumin followed by an analysis of cytotoxicity, LDH enzyme, total reactive oxygen species, antioxidant enzymes (SOD and CAT), fibrosis markers (TGF-β1 protein, fibronectin, and hydroxyproline), and expression of cellular apoptotic markers (Bcl-2, Bax, Bax/Bcl-2 ratio, Annexin V, cytochrome c, and caspase-8). Additionally, the expression of cellular SIRT1 was estimated by ELISA and RT-PCR analysis. Curcumin treatment at doses of 2.7–54.3 µM significantly reduced the growth of sensitive and resistant cells as supported with decreased viability and increased cellular LDH enzyme of treated cells compared to controls non-treated cells. Curcumin also at doses of 2.7 and 54.3 µM regulated the fibrogenesis by reducing the expression of fibrotic markers in treated cells. Analysis of apoptotic markers indicated increased Bax, Bax, Bax/Bcl-2 ratio, Annexin V, caspase-8, and cytochrome c expression, while Bcl-2 expressions were significantly reduced. In curcumin-treated cells at 2.7 μM, non-significant change in ROS with significant increase in SOD and CAT activity was observed, whereas an increase in ROS with a reduction in respective antioxidant enzymes were seen at higher concentrations along with significant upregulation of SIRT1. In conclusion, the present study shows that curcumin induces anticancer activity against resistant cancer cell lines in a concentration- and time-dependent manner. The protective activities of curcumin against the growth of cancer cells are mediated by modulating oxidative stress, regulating fibrosis, SIRT1 activation, and inducing cellular apoptosis. Therefore, curcumin could be tested as an auxiliary therapeutic agent to improve the prognosis in patients with resistant cancers.

## 1. Introduction

Cancer is one of the leading global health problems. Despite advances in cancer research and therapy, the disease’s mortality and incidence have not decreased in the past 30 years [1,2].

Multidrug resistance (MDR) acquired by tumor cells during chemotherapy reduces the efficacy of cancer treatments. Both genetic and epigenetic modifications that accompanied malignancy are involved in MDR. These factors either reduce anticancer drugs’ chemo-sensitivity or block the access of cancer cells to the drugs [3]. Indeed, the use of chemotherapeutic drugs significantly induces the overproduction of different proinflammatory cytokines such as IL-6, chemokines, and anti-apoptotic genes [4]. The molecular link between inflammation and initiation and the progression of oncogenesis was reported through the activation of nuclear factor-kappa B (NF-κB) signaling pathways [5,6], leading to therapeutic resistance [7,8]. Modulation of inflammation and reactive oxygen species (ROS) production were among the promising approaches to reverse MDR [9,10,11].

Natural products are considered rich sources of active constituents with high chemical diversity. These phytochemicals are extensively studied to develop novel therapeutic regimens that can suppress tumor growth and overcome multiple resistance phenomena [12,13].

Curcumin is a commonly used spice that is extracted from Curcuma longa L. rhizomes (Turmeric) in a pure crystalline form [14,15]. According to the US Food and Drug Administration, curcumin is classed as safe for both human consumption and pharmacological purposes without any known side effects [16,17,18].

The bio-functional properties of curcumin and its derivatives—such as anti-tumor, antioxidant, and anti-inflammatory activities [19]—have gained much attention in combating many human diseases, particularly cancer. These properties were attributed to the key elements in the curcumin structure [20]. Curcumin’s natural phenolic, hydrophobic, and antioxidant properties help it to diffuse through cancer cell membranes into the mitochondria, endoplasmic reticulum, and nucleus to perform chemo-preventive, antimetastatic, and anti-angiogenic actions [21,22,23]. Curcumin has been reported to exert its chemotherapeutic efficacy through targeting several molecular pathways involved in mutagenesis, cell cycle regulation, tumorigenesis, apoptosis, and metastasis [16,19,24,25]. Moreover, several studies showed that curcumin significantly reduces fibrosis via suppressing the overproduction of collagen-linked factors such as hydroxyproline, fibronectin, and TGF-β1 in cancer cells [26,27,28,29,30].

In breast cancer, curcumin is shown to suppress the spread of cancer cells to other parts of the body via blocking receptor activator of NF-κB ligand, modulating histone deacetylases (HDAC), histone acetyltransferases (HAT), DNA Methyltransferase 1 (DNMT1), and microRNAs in various breast cancer cells [31,32,33]. In addition, curcumin regulates different signaling pathways, especially signal transducer and activator of transcription 3 (STAT3) [28], cell cycle modulators, and breast cancer gene (BRCA) [33,34], which in turn affects the sensitization of various chemotherapy drugs. In colon cancer, curcumin induces FADD (Fas-Associated Protein With Death Domain), caspase-8, and caspase-3 [35] and inhibits Wnt/beta-catenin signaling, triggering apoptosis [36]. Moreover, it inhibits colon cancer metastasis by P38 MAPK- and JAK/STAT5-dependent suppression of heparanase expression [37]. Similarly, in lung cancer, curcumin modulates the circ-PRKCA/miR-384/ITGB1 pathway, suppressing the malignancy of lung carcinoma. It also decreases matrix metallopeptidase 9 (MMP9) expression, reducing migration and invasion of lung carcinoma [38].

Based on its anti-proliferative efficacy, curcumin has been also studied for MDR modulation in various types of cancer. In this context, curcumin has been reported to sensitize tumor cells to chemotherapeutic drugs and ionizing radiation therapy [14,39,40,41]. Nonetheless, these effects are still controversial [42]. Although, it is reviewed previously in many studies that curcumin reduces the growth and cell viability of different cancer cell lines via oxidative stress and apoptosis [43]. However, little [44] or no data on the role of fibrosis in carcinogenesis and antifibrotic potency of curcumin in cancer cell lines are available, especially with drug resistance. Thus, we proposed that exploring the effect of curcumin on cellular fibrosis might have a good insight into reducing the drug resistance and increasing drug sensitivity of cancer cells by curcumin.

In the present study, we investigated the possible therapeutic role of curcumin in controlling the proliferation of drug-sensitive and -resistant cancers. Thus, the effect of curcumin on oxidative stress, fibrosis, cell proliferation, and apoptosis was evaluated against drug-sensitive (MCF7, HCT116, and A549) and -resistant (MCF7/TH, HCT116R, and A549/ADR) cancer cell lines.

## 2. Results

### 2.1. Cytotoxicity and Inhibitory Effect of Curcumin

**Figure 1:**
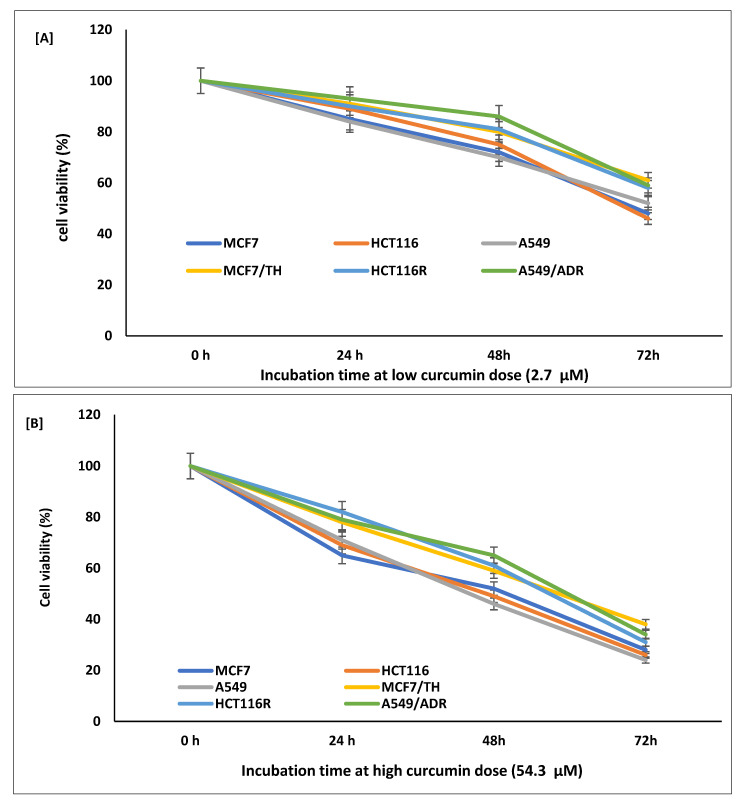
Effect of the time of incubation on the cell viability of both sensitive (MCF7, HCT116, and A549) and resistant (MCF7/TH, HCT116R, and A549/ADR) cancer cell lines treated with curcumin at doses of 2.7 µM (**A**) and 54.3 µM (**B**) concentrations. The results are expressed as the mean ± SD of three independent experiments.

Cell viability was measured by MTT assay. To identify the cytotoxic effects of curcumin, sensitive (MCF7, HCT116, and A549) and resistant (MCF7/TH, HCT116R, and A549/ADR) cancer cell lines were treated at different concentrations of curcumin ranging from 2.7 to 54.3 µM for 72 h. Sensitive and resistant cells exposed to curcumin demonstrated a significant decrease in viable cells in a time- and dose-dependent manner, as shown in Table 1 and Figure 1. At the highest concentration (54.3 µM) of curcumin, the inhibition of cell growth among sensitive cell lines was in the range of (90.5–92.7%), and for resistant cells was in the range of (85.5–87.4%), (Table 1). The results of both lower and higher curcumin concentrations showed significant (*p* < 0.01) inhibition of cell growth in sensitive and resistant cells compared to non-treated curcumin control cells (DMSO, 0.01 *w*/*v*).

In addition, increasing incubation time has shown effectiveness on the inhibitory effect of curcumin. The results in Figure 1A,B showed an increase in the cytotoxicity of curcumin at lower and higher doses in a time-dependent manner. Thus, effective lower (2.7 µM) and higher (54.3 µM) curcumin concentrations were selected for all further biochemical and molecular studies.

### 2.2. LDH Activity

**Figure 2:**
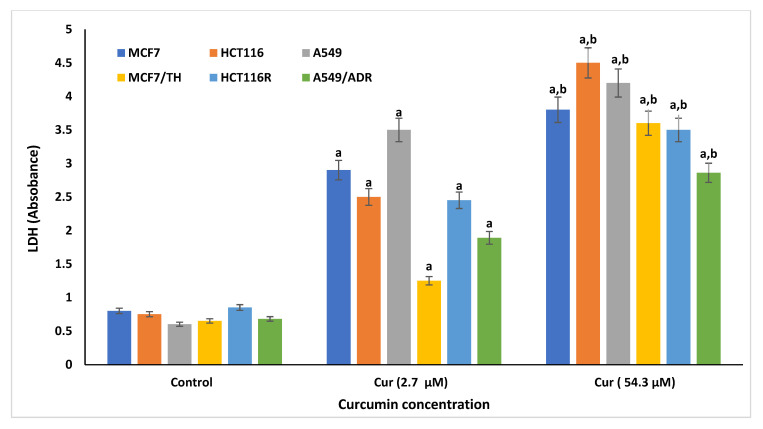
LDH assay revealed cytotoxicity of the curcumin concentration at doses of 2.7 and 54.3 µM against both sensitive (MCF7, HCT116, and A549) and resistant (MCF7/TH, HCT116R, and A549/ADR) cancer cell lines. ^a^ *p* < 0.01, Statistically significant compared to control cells. ^b^ *p* < 0.01, Statistically significant compared to cells treated with 2.7 µM.

Investigation of cytoplasmic enzyme lactate dehydrogenase (LDH) release in vitro from sensitive and resistant cancer cell lines has many advantages and gives extra support for the effective role of the treatment. The released LDH was determined in the supernatant of both curcumin-treated and non-treated sensitive and resistant cells at lower (2.7 µM) and higher (54.3 µM) curcumin doses, Figure 2. Our results showed a higher level of LDH release in treatment groups of sensitive (MCF7, HCT116, and A549) and resistant (MCF7/TH, HCT116R, and A549/ADR) cancer cell lines compared to non-treated control cells (DMSO, 0.01 *w*/*v*).

### 2.3. Effect of Curcumin on the Cellular Reactive Oxygen Species and Antioxdant Enzymes

**Figure 3:**
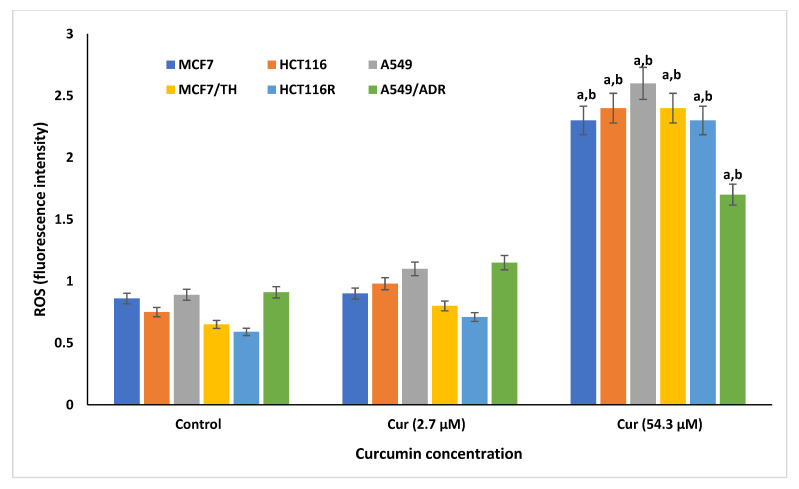
Cellular reactive oxygen species (ROS) produced form all sensitive (MCF7, HCT116, and A549) and resistant (MCF7/TH, HCT116R, and A549/ADR) cancer cell lines treated with curcumin concentration at doses of 2.7 and 54.3 µM, as measured by DCF. ^a^ *p* < 0.01, Statistically significant compared to control cells. ^b^ *p* < 0.001, Statistically significant compared to cells treated with 2.7 µM.

Figure 3 indicates cellular ROS production in control and curcumin-treated sensitive and resistant cancer cells. Cellular ROS was assessed in the supernatant of subjected cell lines treated with curcumin at doses of 2.7 and 54.3 µM. The results showed non-significant increase in the production of ROS from all sensitive and resistant cells at the lower dose of 2.7 µM compared to that of control respective cell lines incubated only on DMSO (0.1% *w*/*v*). In addition, both sensitive and resistant cells treated with curcumin at a dose of 54.3 µM showed an increase in the production of ROS when compared (*p* < 0.001) to cells treated with 2.7 µM, signifying a dose-dependent manner.

**Figure 4:**
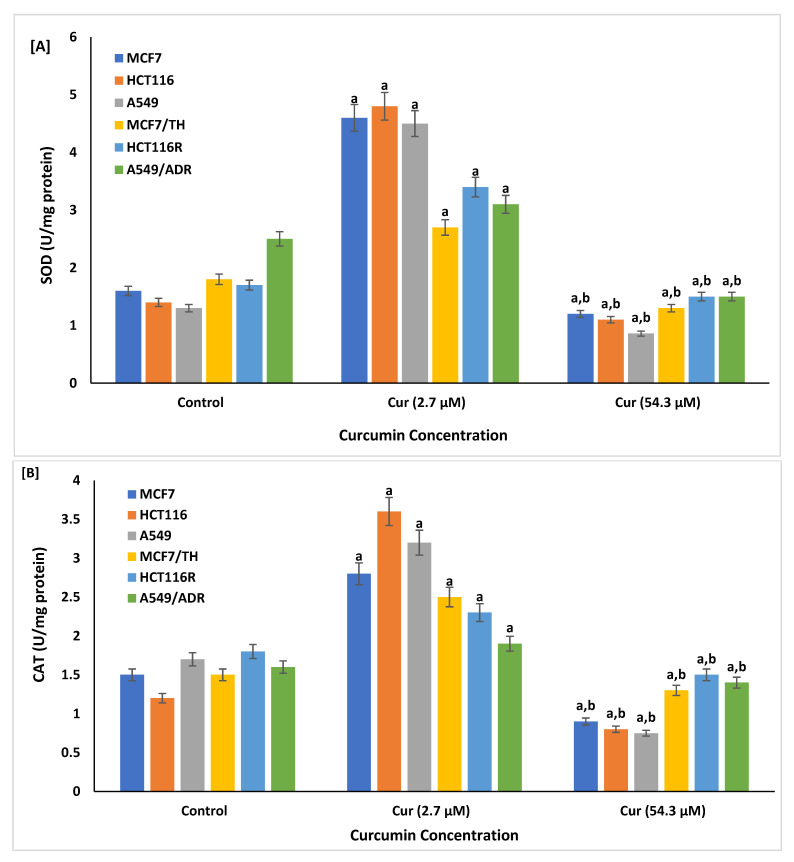
Cellular antioxidant enzymes: superoxide dismutase (**A**,**B**) catalase (CAT) released form all sensitive (MCF7, HCT116, and A549) and resistant (MCF7/TH, HCT116R, and A549/ADR) cancer cell lines treated with curcumin concentration at doses of 2.7 and 54.3 µM, as measured by colorimetric assay. Data are presented as mean ± SD of three independent experiments. ^a^ *p* < 0.01, Statistically significant compared to control cells. ^b^ *p* < 0.001, Statistically significant compared to cells treated with 2.7 µM.

The effects of curcumin at lower doses might have antioxidant properties. The activity of both SOD and CAT were identified as reported in Figure 4. The results showed that both SOD and CAT activity were significantly increased *p* < 0.01 in both sensitive and resistant cancer cells treated with curcumin at a dose of 2.7 µM compared to control non-treated respective cells. However, sensitive and resistant cell lines treated at a dose of 54.3 µM showed lower activity of both SOD and CAT compared to that treated with lower curcumin dose (2.7 µM, *p* < 0.001).

### 2.4. Effect of Curcumin on the Cellular Fibrosis

**Figure 5:**
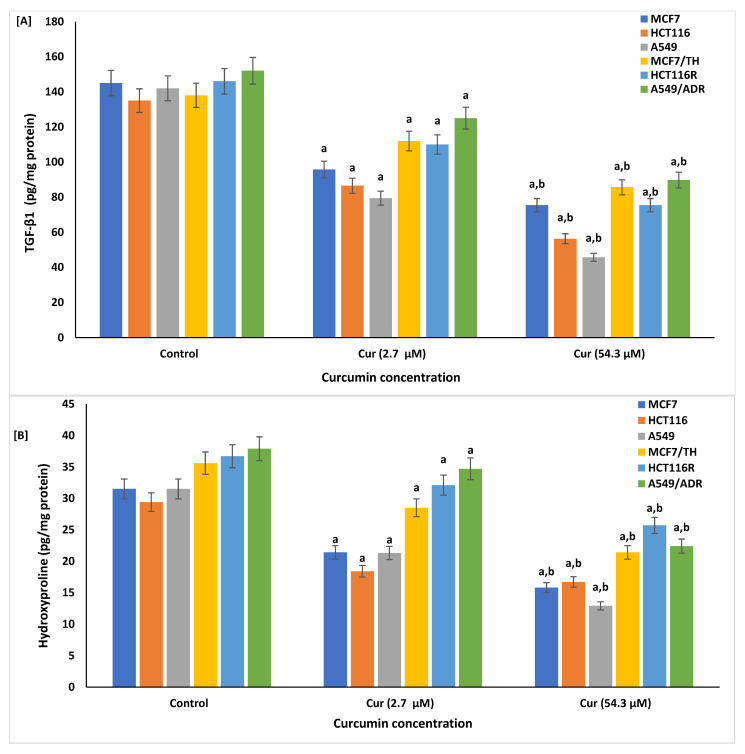

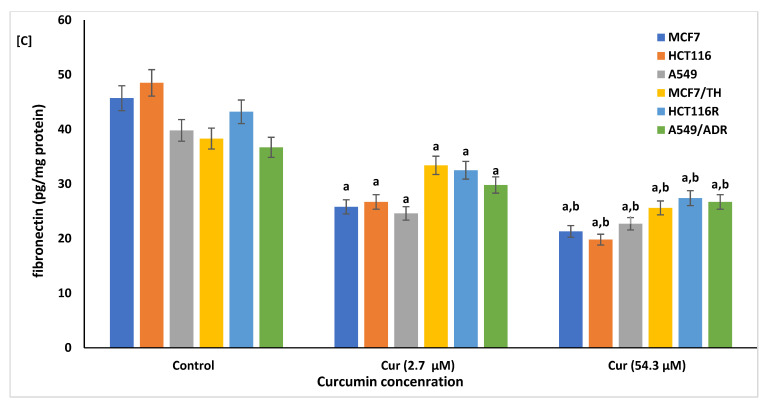
Expression of fibrosis-related markers: TGF-β1 (**A**), hydroxyproline (**B**), and fibronectin (**C**) from sensitive (MCF7, HCT116, and A549) and resistant (MCF7/TH, HCT116R, and A549/ADR) cancer cell lines treated with curcumin concentration at doses of 2.7 and 54.3 µM, as measured by ELISA. Data are presented as mean ± SD of three independent experiments. ^a^ *p* < 0.01, Statistically significant compared to control cells. ^b^ *p* < 0.001, Statistically significant compared to cells treated with 2.7 µM.

Fibronectin, TGF-β1 protein, and hydroxyproline were measured in the drug-sensitive and -resistant cancer cell lines following curcumin treatment at doses of 2.7 and 54.3 µM, Figure 5. The expressions of fibrotic markers, TGF-β1 protein (Figure 5A), fibronectin (Figure 5B), and hydroxyproline (Figure 5C) were significantly reduced following the treatment with curcumin at concentrations of 2.7 and 54.3 µM.

The data show that treatment with curcumin significantly modulates TGF-β and ameliorates the overproduction of fibrosis markers fibronectin and hydroxyproline, suggesting that curcumin can suppress proliferation of tumor cells and invasive cancer cells via antifibrotic action.

### 2.5. Effect of Curcumin on Expression of Sirtuin1 (SIRT1)

**Figure 6:**
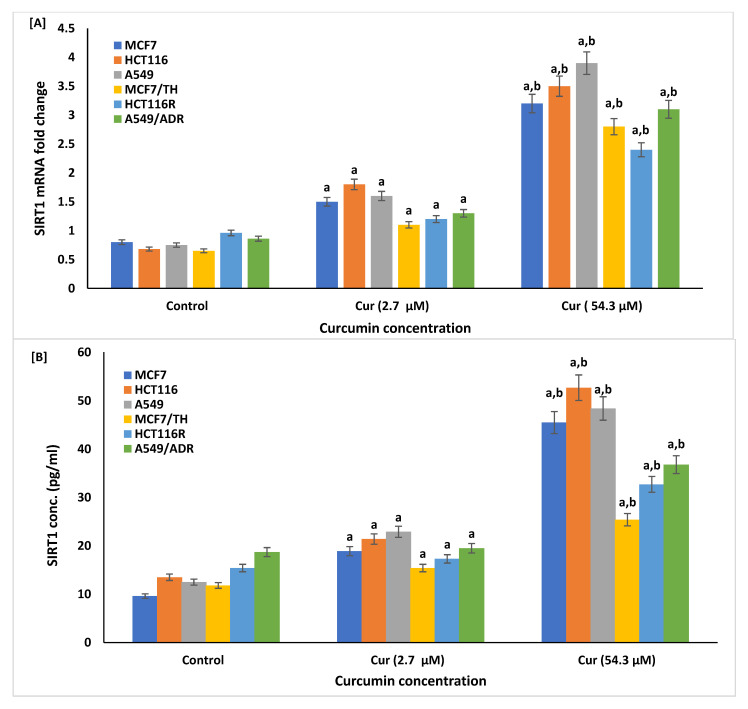
Effect of curcumin (Cur) on the expression of cellular sirtuin1 (SIRT1) in different sensitive and resistant cancer cell lines by real-time quantitative PCR analysis (**A**) and ELISA (**B**). The treatment of cancer cells with curcumin at doses of 2.7 and 54.3 µM significantly increased the levels of SIRT1 mRNA (**A**) and its protein (**B**) in cancer cells compared to control non-treated cells. Data are presented as mean ± SD of three independent experiments. ^a^ *p* < 0.01, Statistically significant compared to control cells. ^b^ *p* < 0.001, Statistically significant compared to cells treated with 2.7 µM.

The expressions of the SIRT1 mRNA and its cellular protein level were identified in sensitive and resistant cancer cell lines treated with curcumin at doses of 2.7 and 54.3 µM using real-time RT-PCR analysis and ELISA, respectively, Figure 6. The results showed that the expression of SIRT1 was significantly amplified in all sensitive and resistant cancer cells following the treatment with curcumin at concentrations of 54.3 µM and significantly amplified in sensitive cells at curcumin at concentrations of 2.71 µM. The upregulation of SIRT1 expression at the genetic or protein level was significantly correlated with cellular oxidation, fibrosis, and apoptosis in sensitive and resistant cancer cells treated with curcumin at lower and higher proposed curcumin concentrations, as shown in Table 2, highlighting the crucial role of SIRT1 in the curcumin efficacy against fibrosis and growth of resistant cancer cells.

### 2.6. Effect of Curcumin on the Cellular Apoptosis

**Figure 7:**
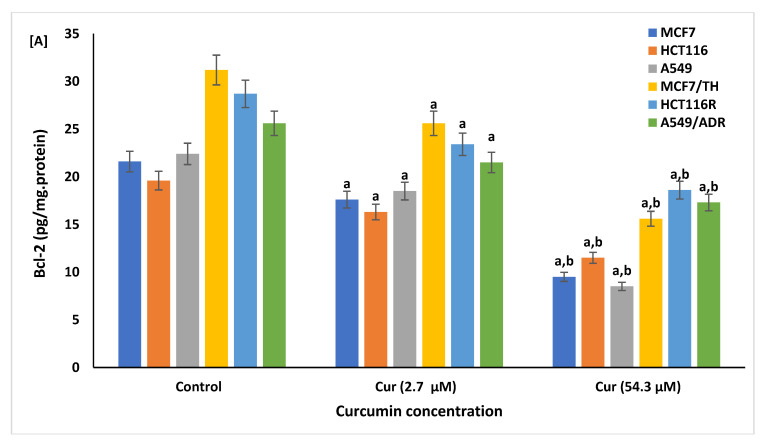

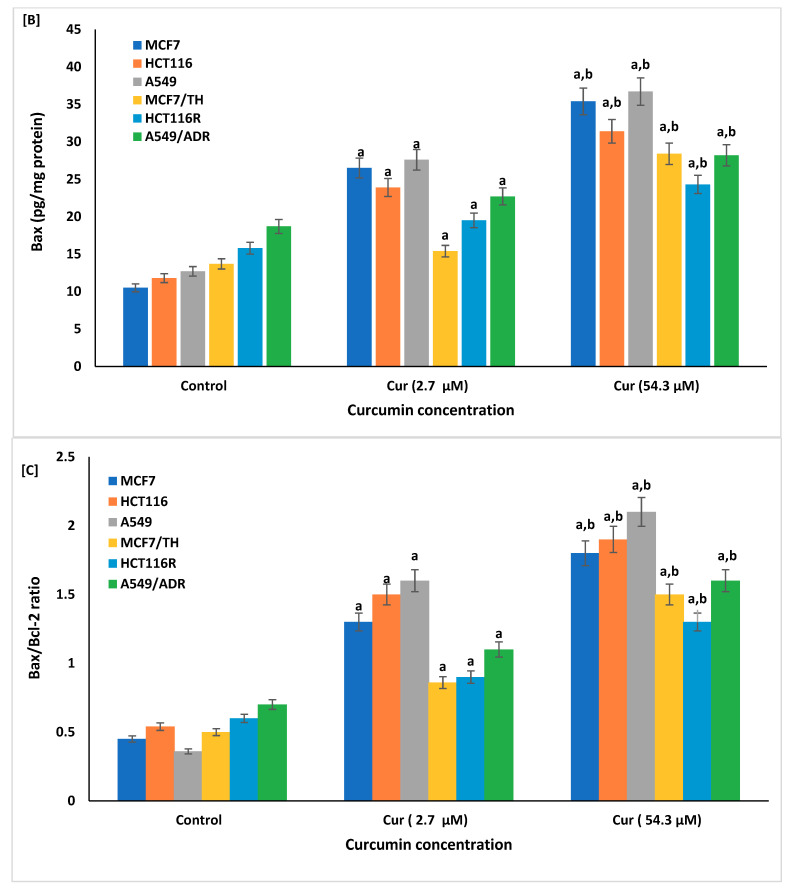

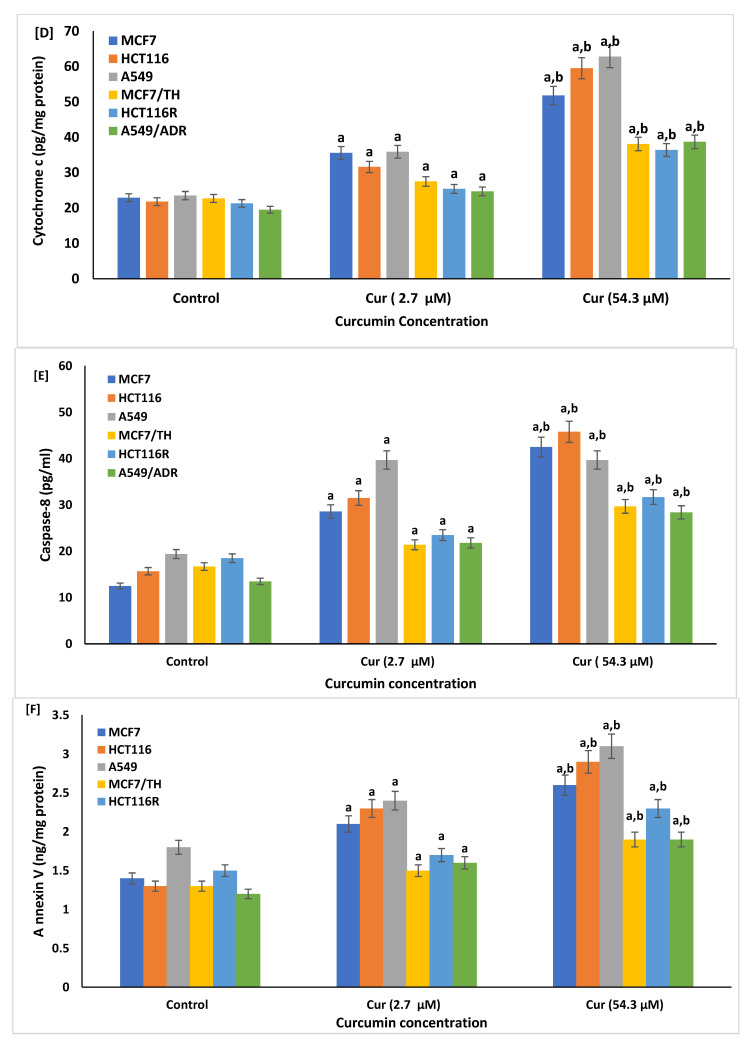
Effect of curcumin (Cur) on apoptotic profiles, Bcl-2 (**A**), Bax (**B**), Bax/Bcl-2 ratio (**C**), cytochrome c (**D**), caspase-8 (**E**), and Annexin V (**F**), in sensitive and resistant cancer cell lines of curcumin-treated and non-treated cells by Cur at doses of 2.7 and 54.29 µM, as measured by ELISA. Data are presented as mean ± SD of three independent experiments. ^a^ *p* < 0.01, Statistically significant compared to control cells. ^b^ *p* < 0.001, Statistically significant compared to cells treated with 2.7 µM.

**Figure 8:**
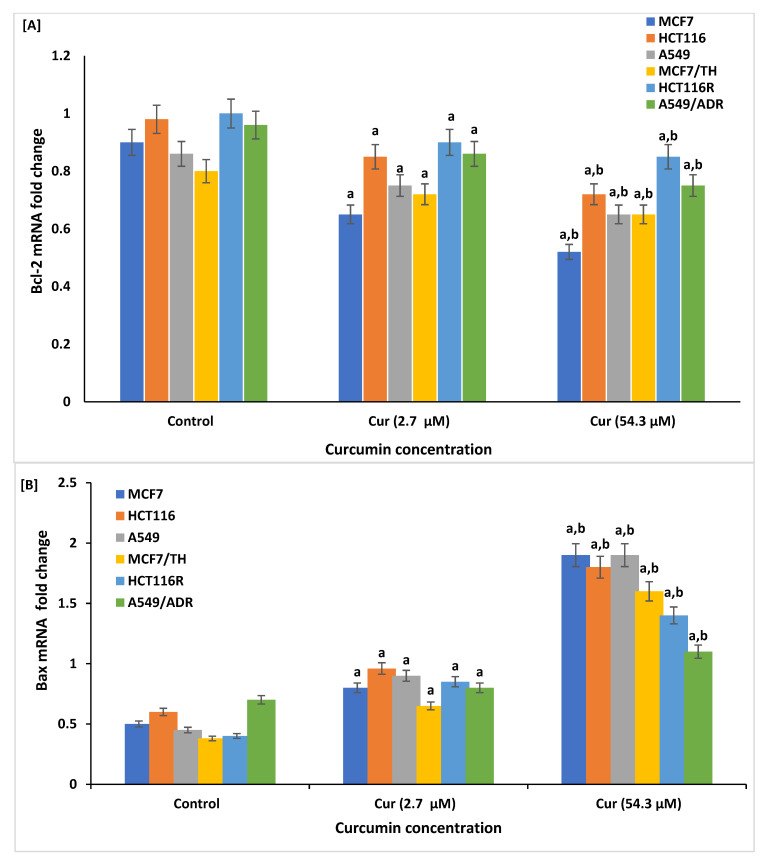

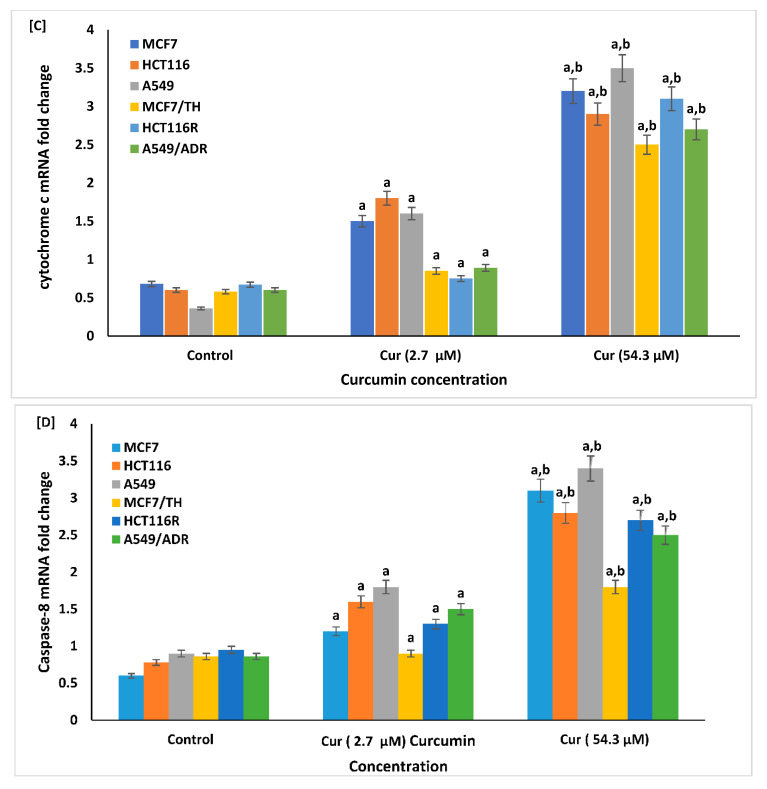
Effect of curcumin (Cur) on apoptotic profiles, Bcl-2 (**A**), Bax (**B**), cytochrome c (**C**), and caspase-8 (**D**) in sensitive and resistant cancer cell lines of curcumin-treated and non-treated cells by Cur at doses of 2.7 and 54.29 µM, as measured by real-time quantitative PCR analysis. Data are presented as mean ± SD of three independent experiments. ^a^ *p* < 0.01, Statistically significant compared to control cells. ^b^ *p* < 0.001, Statistically significant compared to cells treated with 2.7 µM.

The impact of curcumin against cellular apoptosis was estimated in both curcumin-treated and non-treated sensitive and drug-resistant cancer cell lines, Figure 7 and Figure 8. The results of ELISA analysis showed that the genetic and protein expression levels of Bcl-2, Bax, Bax/Bcl-2 ratio, cytochrome c, caspase-8, and Annexin V were significantly changed by curcumin treatment, Figure 7. Compared to non-treated cells, sensitive and resistant cells treated with curcumin at a dose of 2.7 and 54.29 µM showed a significant (*p* < 0.01) reduction in the expression of cellular Bcl-2 (Figure 7A) with higher production (*p* < 0.001) of the proapoptotic proteins, Bax (Figure 7B), Bax/Bcl-2 ratio (Figure 7C), cytochrome c (Figure 7D), caspase-8 (Figure 7E), and Annexin V (Figure 7F) as well. However, sensitive and resistant cells treated with curcumin at doses of 54.29 µM showed significantly more changes of apoptotic proteins compared to cells treated with the lower dose (2.7 µM). These results indicate that curcumin activity against cancer is proceeded by the induction of oxidative stress and apoptosis pathways.

In addition, further supporting PCR analyses for Bcl-2, Bax, cytochrome, and caspase-8 respective genes were performed, Figure 8. The results showed significant reduction in the expression of anti-apoptotic Bcl-2 gene with significant increase in the expression levels of other apoptotic promoting genes, Bax, cytochrome c, and caspase-8 enzyme in cells treated with curcumin at doses of 2.7 µM and 54.29 µM (*p* < 0.01), respectively, as compared with curcumin non-treated cells (control cells, DMSO 0.1%). Similarly, treatment with higher curcumin doses (54.29 µM) showed more significant changes (*p* < 0.001) when compared with lower dose values.

## 3. Discussion

Scientific research on herbal plants and their natural products highlights their use as safer phytotherapeutic agents compared to synthetic drugs. Under biotic and abiotic conditions, medicinal plants produce secondary metabolites with good potency as therapeutic agents against various human diseases, including cancer. It was reported that these metabolites might act as tumor suppressors and apoptotic inducers in cancerous cells [45,46,47,48,49].

For centuries, a hydrophobic polyphenol derivative (curcumin) extracted from the rhizomes of Curcuma longa was applied in food or as a source of traditional remedy in Chinese medicine (CM). In many studies, anticancer efficiency was reported for curcumin alone or in association with other therapeutic agents in many cancers, breast cancer, hepatoma cells, cervical cancer, and oral cancer [50,51,52,53]. In addition, during combination treatments, curcumin has the property of both chemo-sensitization and reversal of the resistance to drugs via the induction of apoptosis [54,55,56].

In this study, curcumin at concentrations of 2.7, 6.8, 13.6, 27, and 54.3 µM significantly suppresses the growth and viability of sensitive (MCF7, HCT116, and A549) and resistant (MCF7/TH, HCT116R, and A549/ADR) cancer cell lines, through activating apoptotic and oxidative stress pathways. Indeed, the inhibition of cell growth at respective curcumin doses for 72 h resulted in a significant decrease in viable cells in a time- and dose-dependent manner.

The release of cytoplasmic enzyme LDH from sensitive and resistant cancer cell lines has been identified as a supportive marker for the effective role of the curcumin treatment. Our results showed a higher level of LDH release in treatment groups of sensitive (MCF7, HCT116, and A549) and resistant (MCF7/TH, HCT116R, and A549/ADR) cancer cell lines compared to non-treated respective control cells. LDH enzyme release following the treatment of cells with recommended curcumin doses is considered as a bio-indicator for cytotoxicity and loss of the cell membrane integrity, confirming the cytotoxic potential of the curcumin at lower and higher doses against the growth of both sensitive and resistant cells. Supporting our results, the LDH enzyme was significantly released into the culture supernatants from the damaged cell membrane of cancer cells treated with various anticancer plant extracts in a manner higher than that of the non-treated control cells [57,58].

Various preclinical research demonstrated promising medicinal properties for curcumin, such as anti-inflammatory, anti-angiogenic, antioxidant, wound healing, anti-proliferative, sensitizer to exert synergistic anticancer activity, and anti-carcinogenic efficacy [59,60,61,62,63]. In addition, curcumin has been reported to promote apoptotic cell death alone or in combination as a sensitizer with other herbs or drugs [54,64,65,66,67,68]. Different concentrations of curcumin exhibited up to 50% cytotoxicity in various cancer models and suppressed the survival growth of cancer cells such as human leukemic lymphocytes and Chinese hamster ovary. In addition, it was reported that at concentrations of 4 and 20 μg/mL, curcumin significantly produced growth arrest and cytotoxicity in various types of cancer [69,70,71].

Many cancer cells can develop resistance to chemotherapy by several mechanistic processes such as epigenetics [72,73], increased drug efflux by ATP-driven drug transporters [74,75], DNA damage repair [76], anti-apoptotic process [77], and epithelial-mesenchymal transition (EMT) [78]. Even at a lower concentration, curcumin significantly leads to promising results against cell viability of MCF-7ADR (±6%) and Tumor Necrosis Factor resistant BT-20TNF breast cancer (8%) cell lines [79,80].

In biological systems, an imbalance in the production of ROS and the antioxidant ability of the cells results in cellular oxidative stress. In aerobic cells, incomplete reduction of molecular O_2_ to H_2_O during mitochondrial oxidative phosphorylation significantly reproduces several ROS-free radicals, particularly superoxide anion (O^2−^), hydrogen peroxide (H_2_O_2_), and hydroxyl radicals (OH^−^) [81]. Similarly, the effect of mechanical chemical and biological stresses, such as exposure to UV rays, ionizing radiation, inflammation, and infection, increases ROS generation [82,83]. However, the baseline levels of ROS activate cell proliferation, survival, differentiation, apoptosis, motility, immune responses, and stress-responsive pathways [84,85].

In the present study, treatment of both sensitive (MCF7, HCT116, and A549) and resistant (MCF7/TH, HCT116R, and A549/ADR) cancer cell lines significantly increased cellular ROS levels in cells treated with a higher curcumin dose 54.3 µM compared to control cells and those treated with a lower curcumin dose 2.71 µM. These results proposed that curcumin might have a bi-functional effect depending on the dose used. This might signify the potential antioxidant activity of curcumin at lower concentrations. In line, the results of antioxidant enzymes’ activity demonstrated that cellular SOD and CAT activity significantly increased in all cancer cell lines treated with a lower dose of curcumin compared to a higher activity reported among drug-sensitive and -resistant cancer cell lines exposed to higher curcumin doses. The increased ROS levels at higher curcumin concentrations inhibit the survival of cell growth and viability of MCF-7/TH, HCT116R, and A549/ADR cancer cells via the triggering of the apoptotic pathways. Many chemotherapeutic agents are known to induce oxidative stress in cancer cells [86]. Low levels of ROS can maintain the stemness of cancer stem cells, contributing to tumorigenesis [57].

Consistent with our results, previous studies reported the dual action of curcumin as an antioxidant [76] and as an ROS-inducing agent to suppress cancer via cell apoptosis [76,87]. In addition, it was reported that curcumin could mediate proapoptotic effects through the generation of more ROS [88,89]. Similar studies also reported that at a 20 μg/mL concentration, curcumin has anti-mutagenic properties [90]. When applied at concentrations over 25 μg/mL, it has clastogenic properties and acts as a pro-oxidant and produces ROS that contributes to apoptosis [91]. Moreover, the presence of turmeric (curcumin) in 2–5% has been reported to be responsible for the protection and suppression of cellular-free radicals ROS, which mediates cellular DNA damage and subsequent cell tumorigenesis [92,93]. The protective role of curcumin against cancer proceeds via modulating several downstream signaling pathways, such as ERK1/2, which is involved in downstream ROS production, suppression of tumor migration, and invasion of cancer cells [94]. Additionally, curcumin was shown to activate endogenous antioxidant enzymes, such as HO-1 and SOD, and suppress the ROS-producing enzyme NADPH: oxidase against tumor invasiveness [95,96].

Overproduction of inflammatory cytokines, fibrotic proteins such as MMP-2 and MMP-9 expressions, and excess extracellular matrix deposition result in fibrosis, which aids in cancer progression [97,98]. The pro-fibrotic cytokine TGF-β is expressed in higher amounts and is known to play a potential role in the pathogenesis of tumors by further increasing other inflammatory mediators involved in tissue remodeling and tumor propagation [99]. Herein, treatment with curcumin significantly modulates TGF-β and ameliorates the overproduction of fibrosis markers fibronectin and hydroxyproline. These data suggest that curcumin at respective doses can suppress the proliferation of drug-sensitive and -resistant tumor cells via antifibrotic action. Increased expression of TGF-β enhances the inflammatory process, fibrosis, subsequent proliferation, and growth of tumor cells [100]. Thus, overproduction of TGF-β induces resistance of cancer cells into chemotherapy [101]. Similarly, TGF-β can stimulate epithelial-mesenchymal transition (EMT) via the upregulation of Smad2/3 [102]. EMT is vital to mechanisms such as inflammation, metastasis of cancer cells, and fibrosis [103,104]. On the other hand, downregulation of the expression of TGF-β remarkably decreases the resistance property, growth, and proliferation of cancer cells [105]. Indeed, treatment with curcumin downregulates the expression of TGF-β1 which in turn inactivates molecular MMP-9 and Smad2 fibrotic markers, suppressing the growth of breast cancer cells [106].

Moreover, many studies showed that curcumin downregulated fibronectin’s mRNA expression and ameliorated the overproduction of fibrosis markers, resulting in the suppression of the growth, proliferation, and colony formation activities in both the MDA-MB-231 and MCF-7 cell lines [107]. Similarly, in leiomyoma cancer, treatment with curcumin significantly reduced the extracellular matrix components and fibronectin production and decreased fibrogenesis and the proliferation of cancer cells [108]. Similarly, our data showed significant downregulation of fibronectin and hydroxyproline in both sensitive and resistant cancer cells by curcumin treatment.

SIRT1, a member of the mammalian sirtuin protein family, is significantly involved in several biological processes, including DNA repair, gene silencing, cell survival, metabolism, and aging [99]. Previously, the role of SIRT1 in fibrosis was supported in several organs such as the liver, heart, and kidneys [100,101].

In this study, the mRNA of SIRT1 and its respective protein were markedly increased in all sensitive and resistant cancer cell lines following curcumin treatment. Treatment with curcumin at doses of 2.71 and 54.3 µM, activates the expression of SIRT1, which is known to significantly suppress the mitochondrial oxidative damage in myocardial ischemia-reperfusion injury [102,103]. Moreover, in rat cortical neurons, curcumin induced SIRT1 activation to inhibit the neurotoxicity of amyloid-β25–35 [104,105]. SIRT1 significantly stimulated ROS formation in ovarian cancer cells [106]. Activation of the SIRT1/NOX4 signaling pathway induces oxidative, apoptotic, and DNA damage in HepG2 cells [107].

In the current study, the potential apoptotic effects of curcumin at doses of 2.7 and 54.3 µM on drug-sensitive and -resistant cell lines were identified. A significant increase in the apoptotic promoting markers, Annexin V, Bax, Bax/Bcl-2 ratio, cytochrome c, and caspase-8, and a reduction in the expression levels of anti-apoptotic marker Bcl-2 were reported in curcumin-treated compared to control cancer cells, indicating an induction of apoptosis in a time- and concentration-dependent manner. Similarly, curcumin exerted anticancer effects by proliferation inhibition and apoptosis induction in breast cancer cells [70]. Curcumin has also been reported to suppress the anti-apoptotic members of the Bcl-2 family and activate the expression of apoptotic-inducing markers: p53, Bax, and procaspases-3, -8, and -9 in colon cancer [108]. The release of mitochondrial cytochrome c also significantly increased in curcumin-treated MCF-7 cells and was found to be linked with the function and expression levels of the Bcl-2 family of proteins [109]. In addition, similar to our results, cellular Annexin V as a marker of apoptosis was significantly increased in cancer cells lines HeLa and HepG2 and normal cell lines BHK and VERO treated with Kalonji extracts (Nigella sativa). In this study, treated cell lines with Kalonji extracts showed increased apoptosis, reduced viability, and proliferation [110].

Finally, the upregulation of SIRT1 mRNA expression significantly correlates with cellular oxidation, fibrosis, and apoptosis in cancer cells treated with curcumin. These results indicate that the SIRT1 pathway plays a vital role in the oxidative stress, antifibrotic, and apoptotic effects of curcumin in cancer treatments. In addition, in our study, curcumin was shown to have a dual effect on ROS generation which is dependent on specific concentrations and seems to induce apoptosis and antifibrosis in sensitive and resistant cancer cells. In conclusion, it is proposed that curcumin could be considered as adjuvant therapy for resistant cancer cells to induce chemo-sensitization effects and direct cancer cells toward apoptosis. However, further studies are needed using curcumin in combination with chemotherapeutic drugs in the studied cell lines.

## 4. Materials and Methods

### 4.1. Reagents

All chemicals used were of analytical reagent grade. Dimethyl Sulfoxide (DMSO), Propidium Iodide (PI), and trypsin were purchased from Sigma (St. Louis, MO, USA). Other cell culture supplies (Roswell Park Memorial Institute (RPMI) 1640 medium, penicillin, streptomycin) were purchased from Gibco BRL (Grand Island, NT, USA). Fetal bovine serum was obtained from Hyclone (Logan, UT, USA), and 3-(4,5-dimethylthiazol-2-yl)-2,5-diphenyl tetrazolium bromide (MTT) was obtained from Fluka (Ron Konkoma, NY, USA).

### 4.2. Preparation of Curcumin

DMSO was used to prepare curcumin (Sigma-Aldrich Corporation, St. Louis, MI, USA, molecular weight; MW= 368.38) at a concentration of 1 mM which was then stored as small aliquots at −20 °C. Curcumin was thawed and diluted as needed in the cell culture medium during cell growth experiments.

### 4.3. Cell Lines

Cancer cells (MCF7, ATCC#HTB-22), colorectal cancer cells (HCT116, Cat. No.: IOC-02P018), and lung adenocarcinoma epithelial cancer cells (A549, ATCC# CRM-CCL-185™) were used as control sensitive cancer cells. MCF7/TH (a multidrug-resistant strain, ATCC, VA, USA, HTB-22™) and colorectal cancer cells (HCT116, ATCC, VA, USA, CCL-247™) were used as drug-resistant cancer cells. In addition, a subline of sensitive A549 cancer cell lines was subjected for measuring its resistant to different concentrations of Adriamycin. The cells were gradually treated with Adriamycin in dose increments from 0.03 μM to 0.5 μM for two weeks for each concentration, as previously reported [111]. After three months, MTT assay was performed and the cells with the highest cell viability were considered the drug-resistant cell line. Then, both the sensitive and drug-resistant cancer cells were treated with curcumin (Supplementary Materials Figure S1).

### 4.4. Cell Culture

In T75 tissue culture flasks, all human cancer cells sensitive (MCF7, HCT116, and A549) and resistant cells (MCF7/TH; a multidrug-resistant strain), HCT116R (5 FU-Resistant colorectal cancer cells), and A549/ADR (Adriamycin-Resistant lung cells) were cultured in RPMI-1640 which were supplemented with 100 μg/mL penicillin, 10% fetal calf serum, 100 μg/mL streptomycin, 20 mM hydroxyethyl piperazine ethane-sulfonic acid, and 2 mM L-glutamine at 37 °C in a CO_2_ incubator. All cells were gradually passaged when they reached 90% confluence and maintained at 37 °C in a humidified incubator with 5% CO_2_.

For activity and maintenance of cultured cell lines, ten thousand cells of each cell line were seeded in triplicates in 6-well plates and passaged every seven days following trypsinization and counting. Both sensitive and resistant cancer cells were subjected to the treatment with different concentrations of curcumin (2.7, 6.8, 13.6, 27, and 54.3 µM) [112].

### 4.5. Cell Viability Assay

Cancer cells were seeded at density of 5 × 10^3^ in 200 μL of RPMI-1640 medium in sterile 96-well plates and cultured overnight, as previously reported [113]. Both sensitive and resistant cells were then treated with a fresh RPMI-1640 media containing the effective lower (2.7 µM) and higher (54.3 µM) curcumin concentrations for 72 h. These doses were selected from cytotoxicity test for all further biochemical and cellular subsequent mechanistic studies. Cancer cells treated with DMSO (0.1% *w*/*v*) were used as control. After that, the cells were incubated with MTT reagent for 4 h by adding a total of 50 μL of MTT (2 mg/mL) to each well. Finally, the cells were treated with 150 μL DMSO for 20 min, and the OD of each well was measured at 490 nm. To lower the test error level, the absorbance of MTT was measured separately in wells without cancer cells and subtracted from the total absorbance. The following two equations were used to calculate the cell viability index [113]:

{Cytotoxicity% = 1 − Absorbance of toxicant/Absorbance of negative control × 100}

{Viability% = 100 − Cytotoxicity%}

### 4.6. Biochemical and Cellular Investigations

All biochemical and cellular investigations were performed at incubation time range 12–48 h, whereas all sensitive and resistant cells entered into exponential growth (log-phase), in which the cell population doubled at a characteristic rate for each cell line. In this phase, the potential effects of drugs, herbal extracts, or even chemical agents which stimulate or inhibit cell growth can be studied [114]. All human sensitive and resistant cancer cells were seeded at a density of 2,000,000 cells per 75 cm^2^ tissue culture flask and incubated at 37 °C and 5% CO_2_ for 24 h to allow for attachment. The cells were then treated with the effective lower (2.7 µM) and higher (54.3 µM) curcumin concentrations for the respective incubation time and centrifuged at 14,000× *g* for 5 min at 4 °C. Finally, the supernatant was transferred to new Eppendorfs and kept on ice until reused for subsequent biochemical analysis.

#### 4.6.1. Lactate Dehydrogenase (LDH) Assay

Cell lines were incubated for 24 h with the effective lower (2.7 µM) and higher (54.3 µM) curcumin concentrations. The supernatant (100 µL) of each culture line was transferred to a 96-well ELISA plate, and the LDH assay mixture (200 µL) (Sigma Aldrich, St. Louis, MI, USA) was added to each well of respective cell line studied. Then, the plate was incubated for 30 min at 25 °C and the intensity of the red color (formazan salt) was developed, indicating LDH release in the treated and untreated cells. Finally, the absorbance as reference of LDH concentration was measured using a microplate reader at 490 nm. The amount of LDH released was reported as the optical density of the control and treated groups.

#### 4.6.2. Assessment of Cellular Oxidative Stress

Cellular ROS was determined by a fluorometric assay with dichloro-dihydro-fluorescein diacetate (DCFH-DA) assay in both sensitive (MCF7, HCT116, A549) and resistant cells (MCF7/TH, HCT116R, A549/ADR) treated with curcumin as previously reported [46]. In brief, 5000 cells were incubated in 96-well plates containing curcumin (2.7 and 54.29 µM) in DMSO for 24 h. Then, the cells were incubated with 25 mM carboxy-H2DCFDA for 30 min at 37 °C, and cellular ROS was measured as fluorescence intensity relative to control at 485 nm excitation and 535 nm emissions. To avoid misreading in cellular ROS, 1 μL of the supernatant of each culture cell line was transferred to a clear 96-well plate containing 100 μL of 1× protein assay solution to measure the protein concentration using the Bradford assay. Finally, fluorescence intensities were normalized with protein concentrations [115].

#### 4.6.3. Assessment of Antioxidant Enzymes

Catalase (CAT) and Superoxide dismutase (SOD) enzymes were identified in all sensitive and resistant cancer cell lines treated with curcumin (2.7 and 54.3 µM) and control cells were incubated in DMSO (0.1% *w*/*v*). For CAT assay, the supernatant of each cell line in a 96-well plate was treated with 12.5 mM KH2PO4 (pH 7.0), 31.25 mM H_2_O_2_ and kept for reaction in light for 45 to 60 s. Then, optical density of all experimental groups contained in wells was measured at 240 nm against a blank. In addition, SOD activity of each cancer cell line was determined by treating supernatants with a reaction mixture consisting of 100 mM KH2PO4 buffer (pH 7.8), 0.1 mM EDTA, 13 mM methionine, 2.25 mM nitro-blue tetrazolium chloride (NBT), and 60 µM riboflavin for 45 to 60 s. The optical density was measured at 560 nm by a spectrophotometer.

#### 4.6.4. Assessment of Cellular Fibrosis

For all curcumin-treated and non-treated sensitive and resistant cell lines, the cell culture medium was centrifuged to remove any contamination, and the culture supernatant was collected for ELISA. TGF-β1 protein levels were evaluated using Human TGF-β1 ELISA Kit (KRISHGEN biosystems, Mumbai, India). In addition, fibronectin and hydroxyproline were estimated by spectrophotometric and immunoassay techniques, as previously reported [116,117,118]. The absorbance (OD) of hydroxyproline in the supernatant of cancer cell lines was determined at wavelength 557 nm using a Systronics-2203 spectrophotometer (Systronics, Gujarat, India) [117]. In addition, the concentration of fibronectin was measured in the supernatants using an immunoassay ELISA kit (ABIN1874233, Atlanta, GA30338, USA) at a wavelength of 450 nm using an ELISA reader (Thermo Scientific™, Cat. No.: 51119000) [118]. For normalization, total protein concentration was performed using Coomassie Plus (Bradford) Assay Kit.

### 4.7. Apoptotic Markers

#### 4.7.1. ELISA Immunoassay Analysis of Bcl-2, Cytochrome c, and Caspase-8

Measurement of apoptotic cellular markers concentration including Bcl-2 having an anti-apoptotic effect (Oncogene Research Products, La Jolla, CA, USA, bcl-2 ELISA, Cat#QIA23), and cytochrome c and caspase-8 having a proapoptotic effect (Zymed^®^Cytochrome c ELISA Kit Cat. No. 99-0040; Bender MedSystems, Vienna, Austria, respectively) were estimated by ELISA according to manufacturers’ instructions.

#### 4.7.2. Assay of Sirtuin1 (SIRT1), Bax, and Anti-Annexin V

Sensitive and resistant curcumin-treated and non-treated cells were assayed for SIRT1 (Eastbiopharm, Hangzhou, USA), Bax (E63; ab32503, Shanghai, China) using commercially available sandwich ELISA kits. In addition, anti-Annexin V as a marker of apoptosis was identified in the lysate of all subjected cancer cells by using immunoassay ELISA kit (Cata. N. BMS252 and BMS252TEN, Thermo Fisher Scientific INC, Vienna, Austria).

### 4.8. Real-Time Quantitative (RT-PCR) Analysis of the mRNA Expression of Apoptotic Markers and SIRT1

#### 4.8.1. RNA Extraction and cDNA Preparation

The mRNA expression of SIRT1, bcl-2, Bax, cytochrome c, and caspase-8 were quantitatively estimated in sensitive and resistant cells treated with curcumin at doses of 2.7 and 54.3 µM [119,120]. This experiment used the RNeasy mini kit (Qiagen, Hilden, Germany) to extract total RNA from the different cancer cell lines. The isolated RNA was treated by RNase free DNase (Qiagen) to remove any genomic DNA contamination. The concentration of extracted RNA was estimated using a Biophotometer (Eppendorf, Hamburg, Germany). According to the manufacturer’s instructions, a total of 100 ng of RNA was reverse transcribed to cDNA by using the RevertAid™ First Strand cDNA Synthesis Kit (Fermentas, Waltham, MA, USA).

#### 4.8.2. Real-Time RT-PCR Analysis of Apoptotic Markers and SIRT1

The Maxima SYBR Green Rox qPCR master mix kit (Fermentas) was used to determine SIRT1, bcl-2, Bax, cytochrome c, and caspase-8 in all cancer cell lines. Real-time PCR reactions were performed using the Steponeplus (Applied Biosystem, Waltham, MA, USA) with corresponding primers of (SIRT1, bcl-2, Bax, cytochrome c, and caspase-8), and standard endogenous control primes of GAPDH (Glyceraldehyde3-phosphate dehydrogenase) (Table 3). The PCR amplification process of corresponding genes was performed for 10 min at 95 °C, followed by 40 cycles of denaturation at 95 °C for 15 s, and finally, annealing and extension steps for 1 min at 60 °C [119,120]. Melting temperatures of specific amplification products and primer dimers were identified from melting curve analysis (60 °C → 95 °C increments at 0.3 °C). These experiments were carried out in triplicate and repeated at least twice. The PCR experiments performed independently were repeated at least three times, and the data obtained were analyzed using the comparative Ct (∆∆ct) method [119,120]. For each cancer cell line, the relative expression level of each of targeted genes was calculated by determining the ratio between the amount of this gene and that of endogenous control.

### 4.9. Statistical Analysis

SPSS statistical program (SPSS, IBM Statistics V.17) was used for data analysis. All the assays were conducted in triplicate, and the results of the continuous variables were expressed as mean ± SD. Two independent sample *t*-tests were used to compare the studied variables, such as DCF-sensitive ROS, LDH, TGF-β1 protein, fibronectin, hydroxyproline, anti-Annexin V, and the expression levels of targeted genes mRNA, SIRT1, Bcl-2, Bax, cytochrome c, and caspase-8 in curcumin-treated and non-treated cancer cells. Pearson’s correlations and multiple stepwise regressions analysis were used to evaluate the association between the expressed SIRT1 mRNA and the studied variables in curcumin-treated and non-treated drug-resistant cancer cell lines. *p*-value of <0.05 was considered statistically significant.

## 5. Conclusions

In conclusion, curcumin induces anticancer activity against sensitive and drug-resistant cancer cells in a concentration- and time-dependent manner. The protective activities of curcumin against the growth of cancer cells are mediated by modulating oxidative stress, regulating fibrosis, SIRT1 activation, and inducing cellular apoptosis. Therefore, curcumin could be tested as an auxiliary therapeutic agent for current treatments to improve the prognosis in patients with drug-resistant cancer.

## Figures and Tables

**Table 1:** In vitro cytotoxic activity of curcumin on sensitive (MCF7, HCT116, and A549) and resistant (MCF7/TH, HCT116R, and A549/ADR) cancer cell lines.

Curcumin Concentrations (µM)	Cell Inhibition/Cytotoxicity (%) on Cell Lines					
	Sensitive Cells			Resistant Cells		
	MCF7	HCT116	A549	MCF7/TH	HCT116R	A549/ADR
2.7 µM	45.6 ^a^	44.3 ^a^	41.5 ^a^	35.8 ^a,b^	37.3 ^a,b^	32.7 ^a,b^
6.8 µM	55.3 ^a^	51.9 ^a^	59.2 ^a^	46.7 ^a^	45.8 ^a^	39.8 ^a^
13.6 µM	62.5 ^a^	68.4 ^a^	67.5 ^a^	56.3 ^a^	59.5 ^a^	53.6 ^a^
27.0 µM	78.6 ^a^	82.5 ^a^	79.4 ^a^	72.8 ^a^	76.4 ^a^	73.3 ^a^
54.3 µM	92.7 ^a^	91.5 ^a^	90.6 ^a^	85.1 ^a,b^	87.4 ^a,b^	86.2 ^a,b^
DMSO (0.1% *w*/*v*) control	N.I.	N.I.	N.I.	N.I.	N.I.	N.I.

Cytotoxicity was measured by MTT assay. IC_50_: half maximal inhibitory concentration, N.I.: no inhibition; ~100% cell growth, DMSO: Dimethyl Sulfoxide. Statistical difference was performed using Student’s *t*-test. ^a^ *p* < 0.01 statistically significant compared to non-treated cells, ^b^ *p* < 0.05 statistically significant compared to corresponding sensitive cells.

**Table 2:** Correlation of sirtuin1 (SIRT1) expression with oxidative stress, fibrosis, and apoptosis markers in curcumin-treated and non-treated cancer cell lines.

Parameters	Control		Treated Cells			
	SIRT1 Activation		SIRT1 Activation			
			Curcumin (2.7 µM)		Curcumin (54.3 µM)	
	R	*p*-Value	R	*p*-Value	R	*p*-Value
Oxidative stress						
Cellular ROS	0.67	0.002	0.69	0.001	0.75	0.001
SOD activity	0.48	0.001	0.34	0.001	−0.82	0.001
CAT activity	0.28	0.001	0.72	0.001	−0.69	0.001
Fibrosis markers						
TGF-β1	−0.52	0.001	−0.89	0.004	−0.96	0.006
Hydroxyproline	−0.84	0.002	−0.72	0.001	−0.85	0.001
Fibronectin	−0.67	0.01	−0.54	0.01	−0.63	0.001
Apoptotic markers						
anti-Annexin V	0.58	0.01	0.38	0.01	0.29	0.01
Bcl-2	−0.35	0.002	−0.41	0.001	−0.56	0.001
BAX	0.65	0.001	0.49	0.001	0.51	0.001
BAX/Bcl-2 ratio	0.85	0.001	0.78	0.001	0.54	0.001
Cytochrome c	0.39	0.001	0.42	0.001	0.61	0.001
Caspase-3	0.58	0.002	0.38	0.002	0.45	0.003

Data are presented as Pearson’s (R) coefficients adjusting for variables identified as cofounders in univariate analyses. SIRT1 mRNA relative expression levels vs fibrosis and apoptotic levels in different sensitive and resistant cancer cell lines following treatment with curcumin at doses of 2.7 and 54.29 µM, respectively, 2-tailed significance. Significance at *p* < 0.05.

**Table 3:** The Primers used for Real-Time Polymerase Chain Reaction.

Primer ID	Primer Sequences
SIRT1 gene	
Forward sequence	5′-TGGCAAAGGAGCAGATTAGTAGG-3′
Reverse sequence	5′-CTGCCACAAGAACTAGAGGATAAGA
GAPDH gene	
Forward sequence	5′-AAGCTCATTTCCTGGTATG-3′
Reverse sequence	5′-CTTCCTCTTGTGCTCTTG-3′
Bcl-2 gene	
Forward sequence	5′-ATCGCCCTGTGGATGACTGAGT-3′
Reverse sequence	5′-GCCAGGAGAAATCAAACAGAGGC-3′
Bax gene	
Forward sequence	5′-ATG GAC GGG TCC GGG GAG CA-3′
Reverse sequence	5′-CCC AGT TGA AGT TGC CGT CA-3′
Caspase-8 gene	
Forward sequence	5′-AGAGTCTGTGCCCAAATCAAC-3′
Reverse sequence	5′-GCTGCTTCTCTCTTTGCTGAA-3′
Cytochrome c gene	
Forward sequence:	5′-AAGGGAGGCAAGCACAAGACTG-3′
Reverse sequence	5′-CTCCATCAGTGTATCCTCTCCC-3′

## Data Availability

The manuscript includes all the data generated or analyzed during this study. Please contact the corresponding author to access the data presented in this study.

## Supplementary Materials

The following supporting information can be downloaded at: https://www.mdpi.com/article/10.3390/life12091427/s1. Figure S1: Experimental methodology.
