# Supplementary figures and images for: Curcumin Modulates Oxidative Stress, Fibrosis, and Apoptosis in Drug-Resistant Cancer Cell Lines
Source: Life (Basel). 2022 Sep 13;12(9):1427. doi: 10.3390/life12091427 (PMC9504331; doi:10.3390/life12091427)

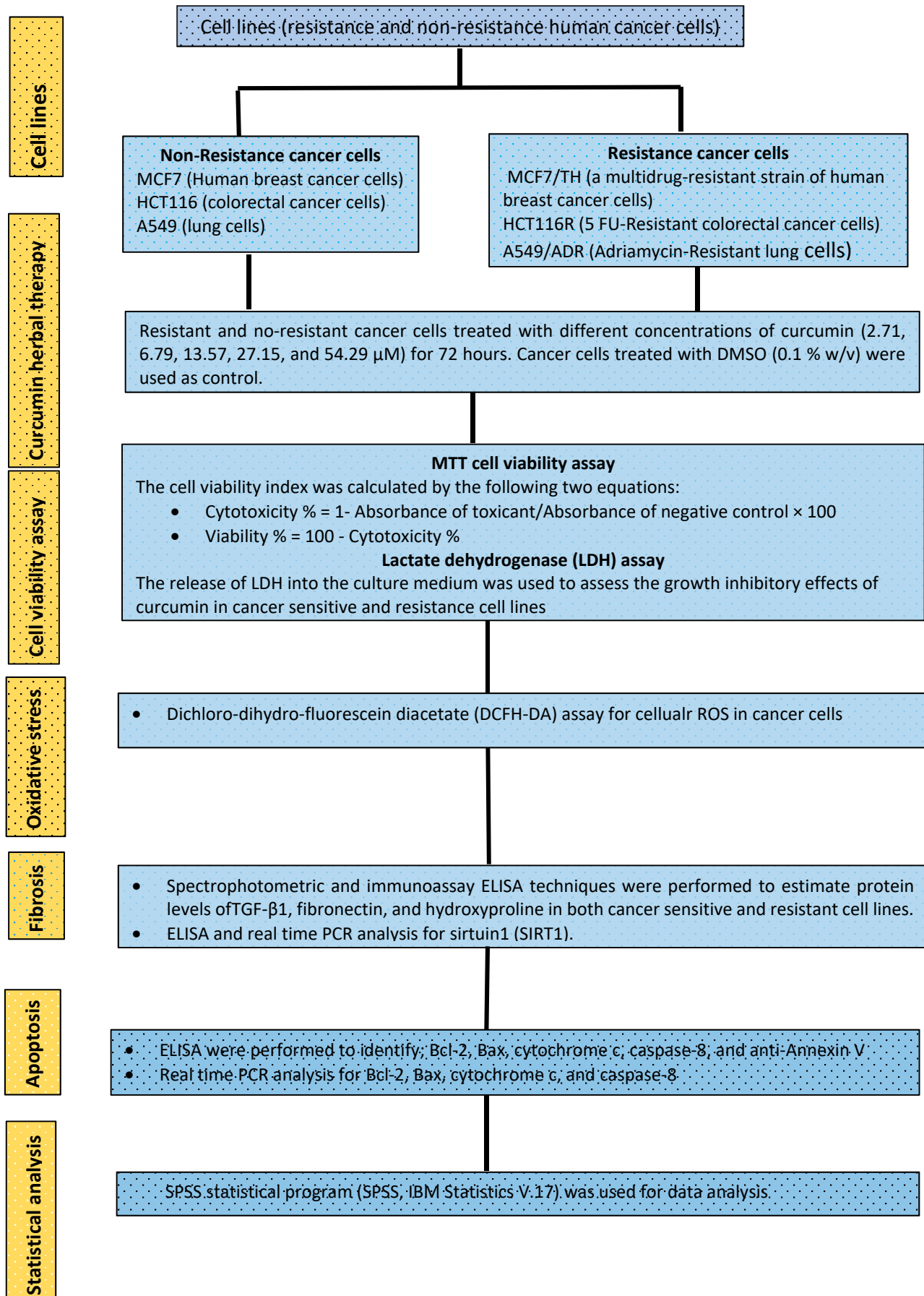

Supplementary Figure S1. Experimental methodology.

Supplement: Supplementary file 1 [file life-12-01427-s001.zip › life-1781124-supplementary.pdf]
